# Determinants of non-adherence to inhaled steroids in adult asthmatic patients on follow up in referral hospital, Ethiopia: cross-sectional study

**DOI:** 10.1186/s40733-019-0053-1

**Published:** 2019-12-30

**Authors:** Bezie Kebede, Girma Mamo

**Affiliations:** 1 Department of Pharmacy, College of Health Science, Mizan-Tepi University, Mizan-Teferi, Ethiopia; 2 Department of Pharmacy, College of Medicine and Health Sciences, Jimma University, Jimma, Ethiopia

**Keywords:** Asthma, Adherence, A steroid inhaler, Outpatient, Referral hospital

## Abstract

**Background:**

Asthma is one of the major non-communicable diseases worldwide. The prevalence of asthma has continuously increased over the last five decades, resulting in 235 million people suffering from it. One of the main challenges in asthma control is adherence to pharmaceutical treatment (4) and leads to poor outcome and increases the economic and clinical burden. Non-adherence could be intentional or non-intentional.

**Objective:**

To identify the determinants of inhaled steroid adherence among adult asthmatic patients.

**Setting:**

The study was done in Jimma university medical center (JUMC) from March–August 22/2018.

**Method:**

Cross-sectional observational study was conducted. Patient assessment was conducted (patient demography, inhalation technique, adherence, and asthma control status). Independent predictors of outcome identified and strength of association between dependent and independent variables determined by using binary logistic regression and statistical significance was considered at *p* < 0.05. Before computing binary logistic regression analysis, the presence of colinearity between independent factor and model fitness was checked.

**Results:**

One hundred forty patients were included in the analysis. Substantial number of patients 53(37.9, 95%CI: 30–45) were non-adherent. Patient experience of previous adverse drug reaction (*p* = 0.011), educational status (*p* = 0.02), patient knowledge status (*p* = 0.028), previous education (*p* = 0.0001) and co-morbidity (*p* = 0.031) were significantly associated with adherence.

**Conclusions:**

The rate of non-adherence to inhalational anti-asthmatics is high and different factors contributed. The health care provider should try to counsel patients about the effect of non-adherence on asthma control. Reassurance concerning adverse drug reactions should be an integral part of patient counseling.

## Introduction

Asthma is one of the major non-communicable diseases worldwide. The prevalence of asthma has continuously increased over the last five decades, resulting in 235 million people suffering from it [1]. The prevalence of asthma in Jimma, Ethiopia was 4.9% [2]. Non-adherence to inhalation therapy is a vital but controllable factor in asthma management that affects disease control. Previous studies showed a high nonadherence rate of 27.2% among asthma patients and different factors such as low Forced Expiratory Volume in 1 s, asthma knowledge and being literate was independently associated with adherence to inhaled corticosteroids [3].

One of the main challenges in asthma control is adherence to pharmaceutical treatment [4] and lead to poor outcome and increases the economic and clinical burden. There is clear evidence of the underuse of asthma controller medications in a prescription receipt, prescription initiation, and medication use once obtained [5].

Non-adherence could be intentional (perceptions of asthma severity, self-manage therapy, fear of side-effects) or non-intentional (forgetful, cost, lack of counseling and misunderstandings) [6].

Asthma has social, clinical and economic impacts on patients, caregivers, and society. The economic burden of asthma is an important measure of its effect on society. Asthma-related costs are high and should be systematically monitored using standardized methods [7]. The study has shown that high acquisition cost of medication, forgetfulness, poor understanding of treatment regimen, fear of side effects of medication, treatment is unnecessary/ineffective, unavailability of drugs and lack of family support and motivation were considered as determinants of medication adherence. Various solutions tried to suggest to overcome this problem: by providing medication reminder tools, by using clinical pharmacist role as an effective tool to counsel and educate the patient regarding the use of medication and drawback of non-adherence, the patient should continuously assed their inhalation technique. Lack of family support might be because of poor knowledge and improving patient and family knowledge about their medication is crucial to improve patient outcomes [8].

In Ethiopia, inhaled corticosteroid is the mainstay of asthma treatment and previous studies showed that adherence is one of the factors in determining asthma control. A various study was done in relation to asthma but there is a lack of adequate data on patient adherence to their inhaled steroids.

The aim of this study was to evaluate risk/determinant factors for non-adherence and provides possible solutions for the existing problems associated with medication non-adherence and optimize treatment outcome/asthma control.

## Method and participants

This study was done in JUMC, Jimma, Ethiopia. JUMC is located in Jimma town which is found in southwest Ethiopia. It is one of the largest governmental established teaching University hospitals in Ethiopia. JUMC is offering diagnosis and treatment for approximately 10,791 patients per month. There are about 9 outpatient clinics located within the hospital which serves over 9592 visits/month [9]. Among these outpatient department (OPD) visits, about 45 patients are asthmatic per month [10]. This study was conducted at OPD service which is a respiratory clinic from March, to August, 22/2018G.C. A hospital-based Cross-sectional study was used to assess determinants of inhaled steroids adherence among adult asthmatic patients attending a respiratory clinic at JUMC. After obtaining the approval from the ethical committee and letter of cooperation from internal medicine the study was initiated at the outpatient medicine department by selecting patients based on inclusion and exclusion criteria. All adults age 18 and above, patients with a confirmed diagnosis of asthma (which is based on Global Initiative for Asthma) and American Thoracic Society (ATS) and have been receiving drugs for asthma from at least last 3 months before the study was initiated. Patients who have followed up at outpatient respiratory clinic were taken as the source population. Newly diagnosed and patients having psychiatric co-morbidity were considered as exclusion criteria. Finally, all adult patients satisfied the inclusion criteria were a candidate as a subject for the study.

### Data collection procedure and data analysis

Socio-demographic data was recorded (age, gender, level of education, occupational status). Medical history (hospitalization for an exacerbation; episodes of progressive increase in shortness of breath, cough, wheezing, chest tightness, or a combination of these symptoms due to different risk factors, duration of illness, co-morbidity; patients having more than one disease concomitantly), smoking status, knowledge (based on bloom’s cut of point included 15 questions, patients having < 60 score were considered as poor knowledge, those having score of 60–80% were moderate knowledge and patients having ≥80% were considered as good knowledge). Attitude (based on Likert scale that contained 12 questions, patients having below mean score were considered as negative attitude otherwise for those above mean score were positive attitude) towards their medication and the disease itself, alcohol and khat consumption, controller therapy (inhaled corticosteroids), inhalation technique (it consists a total of 8 steps and dichotomized to efficient and inefficient based on their performance on critical steps. Patients performed all the three critical steps regardless of the remaining steps were considered as efficient and otherwise inefficient), current medications and adherence (assessed by asthma inhaler test) were recorded using structured questionnaire (adapted from different published literature [11–15]. A questionnaire was translated to the local language and retranslated to English so as to assure consistency.

Relevant patient data was obtained by interviewing the patient and chart review when necessary. An empty and their own MDI was adapted to enable a patient’s inhalation technique to be recorded and they were asked to use their aerosol just as if they would be at home. Before the actual data collection, data collector informed the patient to bring their previous empty MDI device. The principal investigator evaluated the MDI device whether it is empty or not before the patient undertakes it. Adherence was assessed using a 12 item Test of Asthma Inhalation (TAI) that consists of two domains. In the first 10 items, each item scored from 1 to 5 (where 1 was the worst possible score and 5 was the best possible score), with a range from 10 to 50. In addition to ten items, two items also include items 11 and 12 of the health care professional and scored as 1 or 2 (where 1 was bad and 2 was good), with a range from 2 to 4. The second item focus on whether the patient remembers the dose of prescribed medication or not and their efficiency of inhalation technique. Finally, adherence was dichotomized as adherent and non-adherent after summing up the patient score. This validated tool consists of a total of 54 points and patients who got ≥ 50 were considered adherent otherwise non-adherent. Data was collected by one pharmacist after 1-day training about how to extract information from the patient and chart. Supplementary information and clarifications on some patient’s medical information were obtained through discussion with respective health care professionals.

Data were entered using Epi data 3.1software and analyzed with SPSS version 23. All variables were evaluated for normal distribution and found to be normally distributed using descriptive statistics including skewness and kurtosis. Before computing binary logistic regression analysis, the presence of colinearity between independent factor (having less than 2 variance inflation factor) and model fitness (with Hosmer Lemeshow *p*-value 0.13) was checked. Chi-square statistics were used to check the adequacy of cells for binary logistic regression. Independent predictors of outcome and strength of association between dependent and independent variables were identified by using binary logistic regression analysis and *P*-value < 0.25 entered multiple regression. *P*-value < 0.05 was considered as a significant predictor. Descriptive statistics were used to characterize adherence and independent variables. The results of the study were organized in the form of frequencies and percentages. The data was summarized and described using tables and figures.

## Results

### Background characteristics of participants

A total of 140 patients were included in the study. Of which 78 (55.7%) were females. The female to male ratio is 1.25. The overall response rate was about 98%. The mean age was 47.8 (age range 19–74) years with the maximum number of patients being in the age group of 41–59 years. About (32.9%) patients were farmers. The majority of patients 110(78.57%) have no co-morbidity. Hypertension 10(33.33%), diabetes mellitus 9(30.0%) and others account for about 13%. Fifty-seven (40.7%) patients had moderate persistent asthma, 35% had severe persistent and the rest 24.3% had mild persistent. Three (2.1%) of the study subjects are currently smokers. Seventy-one (50.7%) patients were illiterate and only 8(5.7%) attended post-secondary school. Seventy-seven (55%) and 72(51.4%) patients drank alcohol and chew khat respectively. Only 19.3% of patients were efficient in inhalation technique. The majority of patients 88(62.9%) were exposed to biomass fuels during cooking food and other activities shown in (Table 1). The median duration of illness and MDI experience was 4 years (ranges from 4 months-42 years) & 3 years respectively.

**Table 1:** Socio-demographic and clinical characteristics of participants, respiratory clinic, JUMC, Ethiopia, 2018

Socio-demographics and characteristics of patient	Category	Number	Percent	Mean + SD	Range
Sex	Male	62	44.3		
	Female	78	55.7		
Age group	19–40	46	32.85	47.8 ± 15	19–74
	41–59	52	37.15		
	60–75	42	30.0		
Co morbidity	Yes	30	21.43		
	No	110	78.57		
Severity of disease	Mild persistent	34	24.3		
	Moderate persistent	57	40.7		
	Sever persistent	49	35		
Educational status	Illiterate	71	50.7		
	Primary school	30	21.4		
	Secondary school	31	22.1		
	12+	8	5.7		
Residence	Urban	59	42.1		
	Rural	81	57.9		
Exposure of biomas	Yes	88	62.9		
	No	52	37.1		
Knowledge	Good	30	21.4		
	Moderate	47	33.6		
	Poor	63	45.0		
Attitude	Positive	98	70.0		
	Negative	42	30.0		
Smoking status	Non smoker	121	83.43		
	Ex-smoker	16	11.48		
	Currently smoker	3	2.14		
Inhalation technique	Efficient	27	19.3		
	Inefficient	103	80.7		
Do you chew khat?	Yes	60	42.85		
	No	80	57.15		

Fifty-eight (41.42%) patients knew the importance of gargling after inhalation of steroids. Sixty-nine (49.3%) had experienced an asthma exacerbation in the past 12 months. About 29% of patients were admitted to hospital and only 9.76% admitted more than 2–3 times per year.

### Prevalence and reasons for non-adherence

**Fig. 1:**
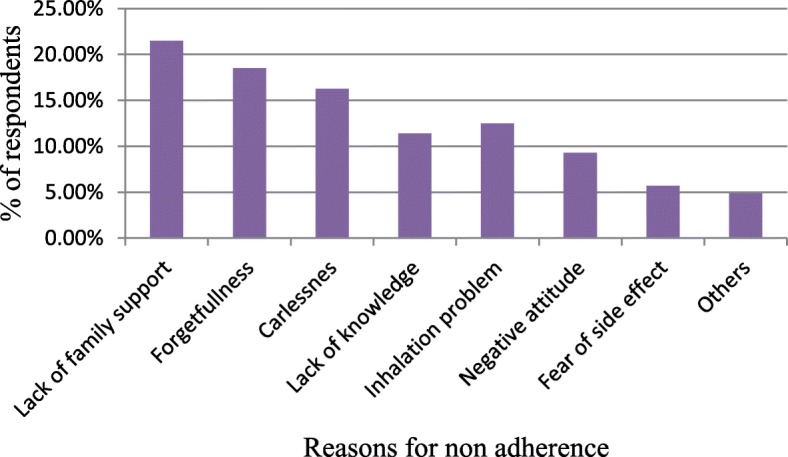
Reasons for non-adherence among participants, respiratory clinic, JUMC, Jimma, Ethiopia, 2018

Eighty-seven respondents were adherent and the rest 53(37.9, 95%CI: 30–45) were non-adherent. A number of factors identified as the reason for non-adherence the patients towards their medication. Lack of family support (those patients who live alone or no family involvement to encourage the patient to take their medication timely) was the major reason for patient non-adherence, 21.5%, followed by forgetfulness, 16.5% and fear of medication side effect shared about 6% (Fig.1). Less than 1% of patients were discontinued their medication in most of their time because they feel better and 1.43% of them missed their medication due to medication acquisition cost is high.

Bivariate analysis showed that male respondents were more likely non-adherent than as compared to females. Regarding educational status, illiterates were more likely non-adherent in comparison with respondents who followed tertiary school. Co-morbidity is one of the risk factors for non-adherence in which patients having co-morbidity is more likely non-adherent as compared to those having isolated asthma. Patients experienced previous adverse drug reaction (patients who developed suspected ADR associated with corticosteroids use previously) and lack of previous education about antiasthmatic medication and their disease were more prone to be non-adherent (Table 2).

**Table 2:** Bivariate analysis for predictors of adherence among asthmatic patients, respiratory clinic, JUMC, Jimma, Ethiopia, 2018

Variables		Non-Adherence (%)		Bivariate analysis	
		Yes	No	COR (CI)	*P*-value
Sex	Male	32(60.37)	30(34.48)	0.3(0.1–0.72)	0.03
	Female	21(39.63)	57(65.52)	1.00	1.00
Age	19–39	17(32.0)	28(32.18)	0.45(0.32–2.1)	0.92
	40–59	13(24.5)	25(28.73)	0.55(0.21–1.45)	.224
	60–75	23(43.5)	28(39.1)	1.00	1.00
Educational status	Illiterate	20(37.73.)	20(23.0)	1.89(1.02–2.54)	0.02
	Primary school	12(22.46)	28(32.2)	0.18(0.063–0.49)	0.1
	Secondary school	17(32.0)	35(40.2)	1.13(0.54–6.1)	0.54
	12+	4(7.63)	4(4.6)	1.00	1.00
Family support	No	32(58.18)	48(56.47)	0.65(0.1–23.76)	0.217
	Yes	23(41.82)	37(43.53)	1.000	1.00
Affordability of drugs	Yes	67(67.0)	19(47.5)	1.9(0.43–12.87)	0.21
	No	23(23.0)	31(52.5)	1.00	1.00
Attitude	Positive	59(75.64)	39(62.9)	1.47(0.622–3.46)	0.38
	Negative	19(24.36)	23(37.1)	1.00	1.00
Previous education	No	53(67.1)	19(31.14)	5.7(5.1–15.3)	.001
	Yes	26(32.9)	42(68.86)	1.00	1.00
Knowledge	Poor	38(55.0)	25(35.2)	3.65(1.303–10.21)	.23
	Moderate	18(26.0)	29(40.84)	2.98(1.203–7.364)	.08
	Good	13(19.0)	17(23.96)	1.00	1.00
IT	Efficient	15(19.0)	17(30.35)	1.4(0.23–2.82)	0.43
	Inefficient	64(81.0)	39(69.65)	1.00	1.00
Previous ADR	Yes	18(34.0)	8(9.2)	6.2(1.8–13.4)	0.012
	No	35(66.0)	79(90.8)	1.00	1.00
Co-morbidity	Yes	21(30.43)	9(12.67)	2.8(1.7–8.432)	0.037
	No	48(69.57)	62(87.33)	1.00	1.00
Asthma control status	Uncontrolled	12(18.46)	6(8.0)	4.21(3.132–11.98)	0.087
	Controlled	53(81.54)	69(92.0)	1.00	1.00

*ADR* Adverse Drug Reaction, *IT* Inhalation Technique, *COR* crude odds ratio

Multivariate analysis was computed to control confounding factors and identify significant determinants of non-adherence. Gender, family support and drug cost were not significant predictors. Illiterate respondents were more likely non-adherent to their medication as compared to respondents who attend post-secondary school (AOR = 2.9, 95%CI: 1.02–5.4, *p* = 0.02). There was a significant association between previous education about the impact of non-adherence and experience to ADR with non-adherence (AOR = 6.3, 95%CI: 7.23–14.3, *p* = 0.0001 and AOR = 3.64, 95%CI: 1.74–11.12, *p* = 0.011 respectively). In this study, non-adherence was significantly associated with patient knowledge (AOR = 2.73, 95%CI: 1.303–10.21, *p* = 0.028) and patients having uncontrolled asthma were more likely non-adherent (AOR = 4.101, 95%CI: 2.13–13.98, *p* = 0.0028) (Table 3).

**Table 3:** Multivariate analysis for predictors of adherence among asthmatic patients, respiratory clinic, JUMC, Ethiopia, 2018

Variables		Non-Adherence (%)		Multivariate analysis	
		Yes	No	AOR (CI)	*P*-value
Sex	Male	32(60.37)	30(34.48)	1.02(0.1–2.2)	0.3
	Female	21(39.63)	57(65.52)	1.00	1.00
Educational status	Illiterate	20(37.73.)	20(23.0)	2.9(1.02–5.4)	0.02
	Primary school	12(22.46)	28(32.2)	0.18(0.063–0.49)	0.1
	Secondary school	17(32.0)	35(40.2)	1.13(0.54–6.1)	0.54
	12+	4(7.63)	4(4.6)	1.00	1.00
Family support	No	32(58.18)	48(56.47)	2.10(1.01–3.76)	0.37
	Yes	23(41.82)	37(43.53)	1.000	1.00
Affordability of drugs	Yes	67(67.0)	19(47.5)	3.9(0.43–12.87)	0.12
	No	23(23.0)	31(52.5)	1.00	1.00
Previous education	No	59(75.64)	39(62.9)	6.3(4.23–14.3)	.0001
	Yes	19(24.36)	23(37.1)	1.00	1.00
Knowledge	Poor	53(67.1)	19(31.14)	2.73(1.303–10.21)	.028
	Moderate	26(32.9)	42(68.86)	1.92(1.03–5.344)	.054
	Good	38(55.0)	25(35.2)	1.00	1.00
Previous ADR	Yes	18(26.0)	29(40.84)	3.64(1.74–11.12)	0.011
	No	13(19.0)	17(23.96)	1.00	1.00
Co-morbidity	Yes	15(19.0)	17(30.35)	2.8(1.7–6.32)	0.031
	No	64(81.0)	39(69.65)	1.00	1.00
Asthma control status	Uncontrolled	18(34.0)	8(9.2)	4.101(2.13–13.98)	0.0028
	Controlled	35(66.0)	79(90.8)	1.00	1.00

*AOR* adjusted odds ratio, *ADR* adverse drug reaction

## Discussion

In Ethiopia, there is no sufficient data concerning inhaler medication adherence and contributing factors. This study was designed to identify determinants of non-adherence to their inhaled steroid medication. Patient adherence to their medication especially for those patients who are on inhaled drugs should be assessed frequently because it has a significant impact on asthma control status. Our study showed that substantial numbers of patients were non-adherent and uncontrolled asthma is significantly more prevalent in non-adherent than in adherents. A similar finding was reported in the previous study done in Jordan [16]. This showed that identification of determinant factors is paramount importance to control asthma, reduce frequent hospitalization/exacerbation, reduce the burden of health care providers and caregivers and reduce health care costs (direct, indirect and intangible costs) and intern to improve quality of life. In Ethiopia, beclomethasone is the only inhaled corticosteroids antiasthmatic drugs. One hundred twenty-two (75.7%) patients were on 2 puff bid whereas the rest 24.3% were on 2 puff/ day.

In our study, the major reported reasons to miss/discontinue medications were lack of family support (21.5%), followed by forgetfulness (16.5%). Being careless about their disease worsening and medication intake was one of the major reasons to stop their prescribed regimen (15.25%). But lack of family support and inefficient inhalation technique were not significantly associated with non-adherent. Other minor reasons were; lack of patient confidence in the treatment regimen and their attitude. The same reason was reported in the previous study [17]. Patients and/ or caregivers should be reminded about their disease and medication use. Health care providers should prepare strategies to minimize/ avoid patient forgetfulness to take their medication.

Identifying risk factors for non-adherence is by far beneficial for the patient to optimize adherence and treatment outcome/asthma control. Our findings showed that different predictors are significantly associated with medication non-adherence. Educational status is one of the major significant predictors for non-adherence.

Illiterate respondents were about three times more likely non-adherent compared to patients attending post-secondary school. A similar result was reported by Janson et al. [18]. Since there are a number of patients who are illiterate and unable to remember what was told by the health care providers, health care providers should continuously evaluate patient compliance.

We also observed a strong association between previous education about the disease/their medication and non-adherence. Patients having previous educational exposure were more likely adherent than who have no exposure. It is consistent with the study reported by Ayele and Tegegn et al. [19] This study suggested that health professionals should counsel the patient during their hospital visit. Williams et al found that patients who received regular adherence feedback from their clinician had sustained levels of adherence >70% compared with those in the control group whose adherence rate fell below 30% [20]It is essential to prepare separate asthma awareness camp for non-adherent patients. Patient education can be organized for noncompliant patients. These patients should be given the opportunity to express their expectations of side effects of medication, disease, response after treatment. Furthermore, the extensive study should be conducted on how to optimize patient adherence.

The previous result reported in Japan, poor adherence to inhaled medicines associated with the complexity of device usage. They found that fewer numbers of inhalations (once inhalation per administration) of selected inhaled medicines was an independent risk factor for poor adherence to patients with asthma [21]. Our study is not consistent with the previous study in which the inhalation technique does not reach statistical significance in determining patient adherence. This could be because of the inhaler device difference in which in our study, the only device used was MDI.

The occurrence of non-adherence to inhaled medications was about three times more likely among patients with poor knowledge in comparing with respondents having good knowledge. The present study suggested that health care providers especially clinical pharmacist should involve patient education programs to boost their knowledge concerning the consequence of being non-adherent and health-related issues to optimize treatment outcomes. This is important to save a patient from unnecessary medication exposure and extra costs associated with dose increment or additional medications.

Upon treatment patients may experienced medication adverse drug reaction and patients having previous adverse drug reaction is three times more likely non-adherent as comparing with patients who have no previous adverse drug reaction experience. Therefore, reassurance about the medication-related problem including minor and major ADR should be the mainstay for health care professionals to combat/minimize non-adherence.

We also found co-morbidity was statistically significant in determining adherence. Patients having isolated asthma were more likely adherent than patients with co-morbidity. This could be the presence of polypharmacy and the pill burden in patients with co-morbidity. Furthermore, the occurrence of nonadherence is higher in those patients having uncontrolled asthma compared with patients having controlled asthma. A similar finding was reported by Ayele and Tegegn et al. [19]. However, sex, age, lack of family support and affordability of drugs were not significantly associated with patient adherence.

### Limitation

This study was conducted in single-center and with a small sample size; difficult to generalize. Adherence was assessed by the self reported way and recall bias may be an issue.

## Conclusion

Despite the improvement of patient counseling habit, the rate of non-adherence to inhaled anti-asthmatics is high. Lack of education about medication, previous ADR and co-morbidities have been identified as statistically significant predictors. More importantly, asthma control status was significantly correlated with non-adherence. Promoting optimal medication adherence strategies are essential to optimize patient benefits. Subsequently, measurement of the degree of non-adherence to the inhaled drug in each individual patient becomes increasingly important in early interventional practice. Pharmacists/and other health care providers should try to counsel patients on the impact of non-adherence on asthma control. Patient reassurance concerning ADR and prevention should be an integral part of patient counseling.

## Data Availability

All data analyzed during this study was available for publication.

## Abbreviation

ADR: Adverse Drug Reaction
IT: Inhalation Technique
JUMC: Jimma University Medical Center
OPD: Outpatient Department
WHO: World Health Organization
